# Combination of ensiling and fungal delignification as effective wheat straw pretreatment

**DOI:** 10.1186/s13068-016-0437-x

**Published:** 2016-01-22

**Authors:** Sune T. Thomsen, Jorge E. G. Londoño, Morten Ambye-Jensen, Stefan Heiske, Zsofia Kádár, Anne S. Meyer

**Affiliations:** Department of Chemical and Biochemical Engineering, Center for BioProcess Engineering, Technical University of Denmark, DK-2800 Kgs, Lyngby, Denmark; Department of Engineering, Biological and Chemical Engineering Section, Aarhus University, Aarhus, Denmark

**Keywords:** White rot, *Ceriporiopsis subvermispora*, Ensiling, Pretreatment, Lignocellulose, Ethanol, Biogas

## Abstract

**Background:**

Utilization of lignocellulosic feedstocks for bioenergy production in developing countries demands competitive but low-tech conversion routes. White-rot fungi (WRF) inoculation and ensiling are two methods previously investigated for low-tech pretreatment of biomasses such as wheat straw (WS). This study was undertaken to assess whether a combination of forced ensiling with *Lactobacillus buchneri* and WRF treatment using a low cellulase fungus, *Ceriporiopsissubvermispora*, could produce a relevant pretreatment effect on WS for bioethanol and biogas production.

**Results:**

A combination of the ensiling and WRF treatment induced efficient pretreatment of WS by reducing lignin content and increasing enzymatic sugar release, thereby enabling an ethanol yield of 66 % of the theoretical max on the WS glucan, i.e. a yield comparable to yields obtained with high-tech, large-scale pretreatment methods. The pretreatment effect was reached with only a minor total solids loss of 5 % by weight mainly caused by the fungal metabolism. The combination of the biopretreatments did not improve the methane potential of the WS, but improved the initial biogas production rate significantly.

**Conclusion:**

The combination of the *L. buchneri* ensiling and *C. subvermispora* WRF treatment provided a significant improvement in the pretreatment effect on WS. This combined biopretreatment produced particularly promising results for ethanol production.

**Electronic supplementary material:**

The online version of this article (doi:10.1186/s13068-016-0437-x) contains supplementary material, which is available to authorized users.

## Background

The amounts of greenhouse gases (GHGs) in the atmosphere recently reached a new record high, which underlines the urgency for action against the accelerating and potentially devastating climate change [1]. Utilization of biomass residues for bioenergy is one potential route to decrease fossil fuel use and in turn decrease GHG emissions [2, 3]. Recently, it has moreover been suggested that the introduction of bioenergy production may enable development in developing countries, e.g. in Africa [4]. When processing lignocellulosic biomass feedstocks, effective pretreatment to decrease the recalcitrance of the biomass is essential. However, currently employed pretreatment methods are targeted towards large-scale systems where economy of scale and efficient throughput generally dictate the technology choice. By contrast, in developing countries, it appears advantageous to produce bioenergy in smaller production units due to the steeply increasing transport costs and the limited biomass logistics and financing options [5–7]. In addition to reforestation and introduction of energy-crop plantations, the wider use of agricultural residues for production of liquid biofuels and implementation of relevant conversion technologies are important in the context of local bioenergy production in e.g. Africa [4]. Such local bioenergy production will invariably be based on low-input agriculture, and low-tech methods will likely be developed first. Biological methods involving white-rot fungi (WRF) or ensiling have recently been investigated for pretreatment of lignocellulosic biomasses such as green grass, wheat straw (WS) and corn stover [7–9]. The results obtained have unequivocally established that none of these biological pretreatments are efficient as standalone pretreatment methods. However, the available data clearly show that such biological pretreatments can function as effective pre-step treatments when combined with other pretreatment methods [9–13]. For example, Ambye-Jensen et al. [9] found that ensiling of WS did not have an effect on enzymatic convertibility on its own, but that ensiling of the straw prior to hydrothermal treatment, lowered the required process temperature in subsequent hydrothermal pretreatment.

Previously, lignocellulosic biomass pretreatments with selected WRF such as *Irpex lacteus* and *Ceriporiopsissubvermispora*, respectively, have been examined. In the case of *I. lacteus* biotreatment, efficient enzymatic conversion of the pretreated material was achieved, but the increased conversion came at the expense of substantial carbohydrate loss during pretreatment [14]. Other studies have found *C. subvermispora* to be among the most promising wild type fungal species for biological biomass pretreatment with respect to improving enzymatic convertibility of glucan in the feedstock [15, 16]. Pretreatment with *C. subvermispora*, expressing only low levels of cellulases, has thus been shown to enhance the biomethane potential (BMP) of different lignocellulosic materials compared to other pretreatments of biomass with other WRF [17, 18]. *C. subvermispora* is apparently able to depolymerize lignin with lower cellulose degradation than other described WRF through a selective delignification process [19, 20], presumably because this fungus lacks or only expresses very low levels of cellobiohydrolases [21]. Tuyen et al. [17] thus investigated the repertoire of genes and their expression patterns in *C. subvermispora* and showed that this fungus has genes to express high levels of Mn peroxidases (up to 13 genes) and laccases (seven genes) but a diminished cellulolytic machinery. Ensiling may facilitate laccase penetration into the lignocellulose complex to enhance lignin degradation [22]. In addition, it has been shown that biological conditioning of biomass can increase the concentration of lignin in hot-water extractives, indicating that ensiling could increase the hydrophilicity of lignin or leave more pores in the fibre wall [23]. This effect might enhance a subsequent WRF pretreatment effect by enabling a better colonization of the lignocellulosic biomass material.

Based on this, we hypothesized that ensiling could be useful as a low-tech method for conditioning biomass prior to WRF pretreatment, and this study was undertaken to investigate whether the combination of forced ensiling with a designated ensiling strain of *Lactobacillus buchneri* and WRF pretreatment with *C. subvermispora* could work as an efficient pretreatment methodology on WS for bioethanol and biogas production. The effects of the combination of ensiling and WRF pretreatment were evaluated with respect to biomass composition, enzymatic convertibility, ethanol and biogas fermentation yields. Moreover, the glucan-, hemicellulose, lignin levels and the changes of extractives content induced by the different pretreatments were assessed.

## Results and discussion

### White-rot fungal pretreatment

**Fig. 1:**
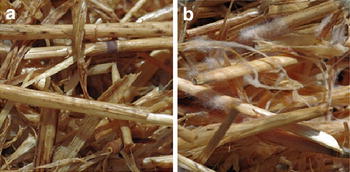
Wheat straw after ensiling and fungal pretreatment: **a** w WS F (visually similar to EWS F and w WS F), **b** w EWS F

WRF pretreatment with *C. subvermispora* was carried out on untreated WS and on ensiled WS (EWS). In order to study the effect of extractives, also washed WS (w WS) and washed EWS (w EWS) samples were included in the set-up. After WRF pretreatment of WS, EWS, w WS and w EWS, it was visually apparent that the w EWS F sample (i.e. ensiled plus fungally pretreated material, which had been washed between the ensiling and the WRF treatment) exhibited the most substantial, uniform and fastest colonization with *C. subvermispora* (Fig. 1).

In general, the WS F, EWS F and w WS F biomass samples were only colonized by the fungus to a very limited extent. On the colonized w EWS F material yellowish droplets were moreover visible. These droplets presumably contained secreted enzymes and metabolized water, and their appearance might be linked to the rapid colonization, since the amounts of secreted enzymes generally correlate to the extent of growth of the fungal biomass [24, 25]. After the WRF treatment, the w EWS F samples had a significantly brighter colour than the WS F, EWS F and w WS F samples. This brighter colour could indicate partial lignin degradation.

During the WRF pretreatments, the total solids (TS) contents remained constant at around 20 % by weight, and none of the samples exceeded a TS level of 23 % at any time, based on gravimetrical measurements. The total TS losses observed during pretreatment were low, the highest TS loss being 5 % by weight in the w EWS F, while being 0, 0 and 2 % in the WS F, EWS F and w WS F, respectively.

The untreated WS generally had very low extractives levels, except that the level of total phenols (0.41 g/100 g TS) was at the same level as that in the other samples (Table 1). Washing of the WS changed the extractives levels very little, although phenols levels decreased. In the EWS extractives, the levels of lactic acid and acetic acid increased as expected and reached 2.1 and 1.5 g/100 g TS, respectively, and also a low amount (0.5 g/100 g) of xylo-oligosaccharides and about 2 g/100 g TS of free xylose were recovered (Table 1)—the free xylose was presumably a residual from the xylose added prior to the addition of the *L. buchneri* preparation used for ensiling (see methods). The washing of the EWS, as expected, reduced the lactic acid and acetic acid levels, and removed about half of the free xylose and about 2/3 of the low amount of free xylo-oligosaccharides (Table 1). It was possible to recover these substances in the washing water (Additional file 1: Table S1). The analysis of the extractives also revealed that the extractives contents of the EWS F sample were similar to those of the EWS sample, underlining that hardly any fungal growth occurred on the unwashed EWS F. By contrast, the w EWS F sample contained relatively high levels of free xylo-oligomers (1.9 g/100 g TS), some gluco- and arabino-oligosaccharides, a very low amount of free glucose, and virtually no free xylose, free lactate or acetate (Table 1). The data, particularly the elevated xylo- and arabino-oligomer levels, indicated that the growth of *C. subvermispora* on the washed ensiled WS was accompanied by liberation of both cellulosic and hemicellulosic oligomers, and thus suggest that the latter might have constituted a significant carbon-source for the fungus during growth. Interestingly, the w EWS and the w EWS F had the lowest levels of soluble phenols among all the samples (Table 1).

**Table 1:** Soluble carbohydrates, acids and phenols in water extracts of the differently pretreated samples (the contents of xylitol and formic acid were below 0.1 g/100 g TS in all samples, data not shown)

g/100 g TS	WS	EWS	w WS	w EWS	WS F	EWS F	w WS F	w EWS F
Free Sugars								
Glucose	0.16 ± 0.02	0.00 ± 0.00	0.00 ± 0.00	0.00 ± 0.00	0.00 ± 0.00	0.00 ± 0.00	0.09 ± 0.14	0.24 ± 0.02
Xylose	0.07 ± 0.06	2.10 ± 0.04	0.02 ± 0.00	0.95 ± 0.05	0.01 ± 0.01	1.89 ± 0.13	0.08 ± 0.08	0.01 ± 0.02
Arabinose	0.00 ± 0.00	0.00 ± 0.00	0.00 ± 0.00	0.00 ± 0.00	0.00 ± 0.00	0.19 ± 0.02	0.00 ± 0.01	0.00 ± 0.00
Oligomers								
Glucan	0.12	0.11	0.13	0.05	0.26	0.22	0.49	0.45
Xylan	0.25	0.46	0.14	0.16	0.72	0.63	0.97	1.89
Arabinan	0.03	0.06	0.02	0.03	0.18	0.12	0.23	0.53
Acids								
Lactic acid	0.00 ± 0.00	2.10 ± 0.08	0.01 ± 0.00	0.60 ± 0.02	0.01 ± 0.01	1.91 ± 0.04	0.00 ± 0.00	0.00 ± 0.00
Acetic acid	0.16 ± 0.02	1.49 ± 0.04	0.09 ± 0.00	0.37 ± 0.01	0.03 ± 0.02	1.22 ± 0.09	0.02 ± 0.01	0.05 ± 0.00
Total phenols	0.41 ± 0.05	0.22 ± 0.02	0.23 ± 0.02	0.11 ± 0.03	0.57 ± 0.05	0.39 ± 0.03	0.57 ± 0.05	0.14 ± 0.02

Standard deviations are presented when applicable

The analysis of the solid fractions, after the different pretreatments, showed that the w EWS F samples had a lignin content of only 13.5 ± 0.6 g/100 g TS, while the lignin contents in all other samples were 19–22 g/100 g TS (Table 2). The latter data indicate that there was a lignin-degrading effect of the *C. subvermispora* WRF pretreatment of the ensiled, washed, WS. The mass balances for the TS of the pretreated samples did not all close at 100 % (Table 2) making it difficult to directly estimate the significance of this apparent delignification. However, when assessing the resulting effect on the relative glucan enrichment of the biomass, i.e. by comparing the ratio glucan:[glucan + xylan + arabinan + lignin] in the samples, it is evident that the observed decrease in lignin in the w EWS F samples, produced a relative enhancement of the glucan, by increasing the glucan:[glucan + xylan + arabinan + lignin] ratio to 0.56 in the biomass, whereas this ratio was ≤0.51 in all the other samples (Table 2) (Compared with the glucan enrichment on the basis of glucan/[Sum of all components], i.e. including ash and extractives in the denominator, the glucan enrichment in the w EWS F samples was 0.48 vs. a value of ≤0.42 in the other pretreated samples). This relative increase in glucan in the solid fibre fraction is presumed conducive to achieving improved enzymatic conversion of the carbohydrates in the fibre fraction (see next section). A delignification effect has also been seen in previous studies using *C. subvermispora* for WRF pretreatment [6, 15, 26], but the results have not always been unequivocal. For instance, Cianchetta et al. [15] investigated lignin reduction of WRF pretreated WS with *C. subvermispora*, but did not observe any significant lignin reduction after 4 weeks. However, after 10 weeks of the fungal growth, a lignin reduction similar to the one in our study was observed, but the lignin reduction came with a TS loss of 20 % [15].

**Table 2:** Composition of ensiled and WRF-pretreated biomasses and controls

g/100 g TS	Glucan	Xylan	Arabinan	Lignin	Ash	Extractives	Sum	G/G + X + A + L
WS	38.0 ± 1.1^c^	20.1 ± 0.5^b^	2.4 ± 0.3^cd^	20.7 ± 0.2^ab^	5.0 ± 0.1^a^	3.4 ± 2.1^b^	89.5	0.47
EWS	36.0 ± 1.7^cd^	20.1 ± 0.6^b^	2.6 ± 0.2^bc^	19.5 ± 0.6^ab^	3.6 ± 0.2^ab^	7.1 ± 1.4^a^	89.0	0.46
w EWS	47.8 ± 1.5^ab^	26.4 ± 1.0^a^	3.3 ± 0.3^a^	19.5 ± 0.4^ab^	3.3 ± 0.2^ab^	1.3 ± 2.5^b^	101.7	0.49
w WS	49.5 ± 0.3^a^	25.1 ± 0.5^a^	3.1 ± 0.3^ab^	20.3 ± 1.1^ab^	3.7 ± 0.2^ab^	3.0 ± 1.4^b^	104.8	0.51
WS F	34.1 ± 3.0^cd^	17.0 ± 1.9^cd^	2.0 ± 0.3^de^	20.5 ± 0.3^ab^	4.9 ± 0.4^ab^	2.6 ± 0.9^b^	81.1	0.46
EWS F	31.6 ± 3.0^d^	17.1 ± 1.2^cd^	2.1 ± 0.2^cde^	19.3 ± 0.3^b^	4.2 ± 0.8^ab^	5.9 ± 2.8^ab^	80.1	0.45
w WS F	32.3 ± 0.3^d^	15.4 ± 0.1^d^	1.6 ± 0.0^e^	21.8 ± 1.3^a^	4.2 ± 0.5^ab^	3.6 ± 1.2^b^	79.0	0.45
w EWS F	43.6 ± 1.4^b^	19.1 ± 0.7^bc^	2.1 ± 0.3^cde^	13.5 ± 0.6^c^	3.4 ± 0.9^b^	8.3 ± 1.3^a^	90.1	0.56

Data are given as average values ± standard deviation. Different roman superscript letters indicate significantly different average values in the same column (*p *< 0.05). G/G + X + A + L is an estimate of relative glucan enrichment of the biomass, calculated from the g/100 g TS values as Glucan/(Glucan + Xylan + Arabinan + Lignin)

The quantitative data for the extractives of the solid fractions came with relatively high standard deviations, and the absolute values were low, ranging from 1.3–8.3 g/100 g TS (Table 2). It is nevertheless tempting to speculate that in addition to the ensiling treatment itself, presumably inducing a mild pretreatment effect of the glucan by the acids produced [9], the removal of the soluble matter/extractives from the EWS by the washing facilitated the WRF growth. The w EWS samples thus had the lowest measured level of extractives (1.3 g/100 g TS, Table 2), whereas the levels in the EWS samples were higher (7.1 g/100 g, Table 2). Apparently, the fungal growth in turn also produced some extractives, since the levels in the w EWS F were higher than in the other samples (8.3 g/100 g, Table 2).

Analyses of hydrophilic extractives in WS have shown that phenolic substances, fatty acids and sugar alcohols are dominant substances [23], but sterols, waxes, steryl esters and triglycerides have also been reported to be present in hot-water extractions of WS [27]. For several hardwood species, it has been found that the extractives content are related to durability against fungal attack [28], and although no direct correlation has been reported, hardwood extractives are known to inhibit the growth of some WRF under laboratory conditions [29]. However, the response of *C. subvermispora* to phenolics, including those likely encountered during lignin modification/degradation, is highly complex, and appears to be compound specific with respect to both growth and enzyme production. *C. subvermispora* has thus been found to produce increased lignin-modifying enzyme levels (laccase and manganese peroxidase) in response to, e.g. syringic acid and other di-methoxylated compounds that might occur as soluble substances during white-rot growth on lignocellulosic biomass [30].

### Enzymatic convertibility

**Fig. 2:**
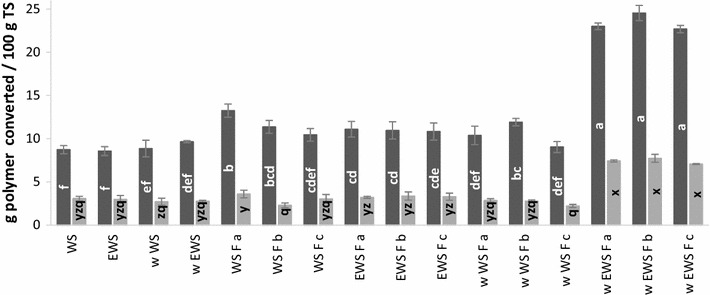
Enzymatic convertibility of carbohydrates of WRF-pretreated material in triplicates (a, b and c) and raw material. *Dark grey* glucan, *light grey*: xylan, arabinan were below 0.5 g/100 g TS in all samples. The results are grouped according to significance (*p* = 0.05 %), where ‘a’ is significantly higher than ‘b’ and so forth. *Error bars* represents standard deviations

The efficiency of the pretreatment was evaluated by enzymatic convertibility. The enzymatic convertibility was determined on each of the triplicates of the WRF pretreatments. The results obtained for w EWS F were comparable for the triplicates (not different, *p* >0.05 % for both glucan and xylan), which confirmed the reproducibility of the pretreatment method (Fig. 2). The enzymatic convertibility of the samples that were not subjected to fungal pretreatment (WS and EWS) are comparable to results from our previous work on ensiling of WS [9].

It is clear that w EWS F produced significantly higher enzymatic convertibility of both glucan and xylan, compared to the remaining fungal pretreatment as well as the raw materials. On average 23.4 ± 1.1 g glucan and 7.4 ± 0.4 g xylan were enzymatically converted in w EWS F per 100 g TS. The convertibility of the w EWS F was approximately twice as much as that achieved in the other samples and three times as much as that obtained for the untreated straw (Fig. 2) (For the w EWS F the convertibility was equivalent to a ratio of glucan:xylan conversion of 3.2, whereas the converted glucan:xylan ratio of untreated WS was 2.9 as calculated from the data in Fig. 2). Apparently the washing step helped support the colonization of *C. subvermispora* in the w EWS F biomass. Furthermore, there was no corresponding sugar release of the w WS, i.e. washed straw, but not subjected to ensiling. The fact that the enzymatic convertibility of both xylan and glucan was improved in w EWS F corroborated that there was a true and significant pretreatment effect and not only an effect of an up-concentration of glucan (Fig. 2). The attained level of 23.4 ± 1.1 g glucan converted/100 g TS is comparable to previously published results of 22.8 ± 1.9 g glucan converted/100 g TS obtained for classically hydrothermally pretreated WS (180 °C for 10 min) [9].

### Ethanol fermentation

**Fig. 3:**
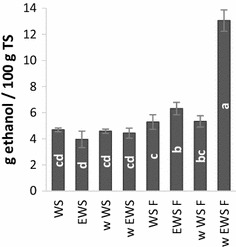
Simultaneous saccharification and fermentation of pretreated materials and controls. The results are grouped according to significance (*p* = 0.05 %), where ‘a’ is significantly higher than ‘b’ and so forth. *Error bars* represents standard deviations

The pretreatment effect was confirmed in a simultaneous saccharification and fermentation (SSF) assessment where w EWS F produced an increase in ethanol yield of 278 % compared to untreated WS (Fig. 3). A final yield of 13.1 ± 0.8 g ethanol/100 g TS was reached, corresponding to 66 % of the theoretical maximum based on glucan content (Fig. 3). This ethanol yield is comparable with published results on high-tech pretreatment of WS [9, 31, 32]. For example, Thomsen et al. [31] reached similar ethanol levels when using a three-step hydrothermal treatment reactor system in pilot plant scale. Thereby, the validity of the combined ensiling and fungal biotreatment approach as potential low-tech pretreatment method is verified, especially when taking into account that neither the enzymatic conversion nor the fermentation were optimized. Zeng et al. [33] have shown that the biological pretreatment performance on WS can be greatly enhanced in the presence of inorganic salts. A similar approach might increase the effect of the tested WRF pretreatment in future set-ups.

The fermentations showed no sign of inhibition in the gravimetrical monitoring (data not shown), not even in the very early stages of fermentation. Formation of inhibitors is linked to elevated pretreatment temperatures commonly employed in biomass pretreatment, where a wide range of sugar degradation products may form [34]. The avoidance of such inhibitor formation effects is an additional positive trait of WRF pretreatment. Furthermore, after WRF pretreatment, the pH of the wet pretreated biomass was nearly optimal for SSF (pH ~ 5) (data not shown). This pH is more optimal for further enzymatic processing than that obtained after common pretreatments such as hydrothermal (pH ~ 3–4) or alkali pretreatment (pH > 9). Therefore, there is no need for using large quantities of chemicals (such as lime) for pH adjustment prior to the fermentation when using the presented combination of biopretreatment.

Even though there are perspectives for the presented combination of biopretreatments as a low-tech pretreatment option, there are still important issues to address: sterilization of the biomass prior the fungal inoculation was needed in order to be able to differentiate between the effect of the fungal treatment and that of other microbes for the research. In a previous study, low doses (in the order of ppms) of inexpensive chemicals such as sodium sulphate were used to delay microbial growth while *C. subvermispora* colonized the biomass [35]. Alternatively, a short steaming procedure at ≤100 °C has previously been sufficient to allow *C. subvermispora* colonization without compromising its delignification abilities [36]. Similarly, it is possible to design the washing step in-between the ensiling and WRF inoculation to favour *C. subvermispora* colonization.

In general, this work has addressed the use of a low-tech, inexpensive pretreatment for small-scale units. Scale-up of the procedure is an obvious challenge, which has not yet been accomplished on WS. However, within the pulping industry, biopulping trials (with WRF) have been executed with wood chips on 50-ton pilot scale with satisfactory outcomes [21, 37], suggesting a potential for successful scale-up of the WRF pretreatment of WS as well as other lignocellulosic feedstock. Therefore, the presented approach offers opportunities for biomass pretreatment in cases where external factors require low process complexity and small-scale implementation as, e.g. in the developing world.

### Biogas production

**Fig. 4:**
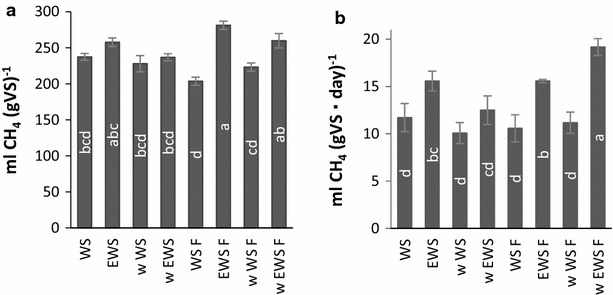
**a** BMP of pretreated samples and controls. **b** Biogas production rate first week of production. The results are grouped according to significance (*p* = 0.05 %), where ‘a’ is significantly higher than ‘b’ and so forth. *Error bars* represents standard deviations

The positive effect of WRF pretreatment seen in enzymatic convertibility and fermentation for the w EWS F samples did not produce higher increases in the biomethane potential (BMP) than the other ensiled samples after anaerobic digestion (Fig. 4a). The ensiled samples, EWS, EWS F, w EWS and w EWS F tended to have higher BMP than the non-ensiled samples, but the EWS F and EWS samples reached the same BMP level as the w EWS F (Fig. 4a). Presumably, the reason for the similarities among the ensiled, but otherwise differently pretreated samples, may be related to the anaerobic digestion process, which utilizes a large range of organic compounds, i.e. sugars as well as fatty acids and proteins. This utilization means that the conversion is not necessarily limited by the accessibility of the glucan. The overall higher BMP of the ensiled samples can be explained by the availability of organic acids resulting from the ensiling process, but may also relate to any possible physical changes of the biomass resulting from the acidic ensiling, such as decreased hydrophobicity enabling better suspension of solids, despite no profound chemical compositional effects being evident.

In contrast, evaluation of the biogas production *rate* during the first week of production clearly showed that the initial conversion rate of the w EWS F sample was higher than that of the other samples, a result that we interpret as being ascribable to the organic compounds of the biomass likely being more accessible to the fungus after ensiling and in turn enabling faster growth and conversion (Fig. 4b). Thus, the higher initial biogas production rate and the relatively high BMP of the w EWS F sample corroborated the efficiency of the w EWS F pretreatment compared to the w EWS treatment. Based on the anaerobic digestion alone, it can be argued that the combination of biopretreatments presented in this study has an insignificant effect on the BMP of WS. Nevertheless, the faster conversion may potentially translate into options for using smaller, and thus cheaper, digesters.

## Conclusions

This study demonstrated that a substantial colonization of *C. subvermispora* in WS is facilitated by a combination of ensiling and a washing step as preconditioning, resulting in a pronounced pretreatment effect. The combination of ensiling and washing removed extractives such as waxes, fats and toxic compounds, which in turn, permitted the subsequent fungal growth and delignification. The combination of ensiling, washing and fungal growth produced efficient pretreatment of WS as assessed by enzymatic convertibility and ethanol yields. Furthermore, the results were achieved with only a minor TS loss due to fungal metabolism. When assessed for biogas production, the initial biogas production rate was significantly increased by the combination of biopretreatments, while the biomethane potential of the WS was not significantly improved.

In the present study, both the ensiling and the WRF pretreatment were done under controlled conditions employing defined cultures for each step. In the context of use in developing countries, it is likely necessary to establish some microbiological routines to maintain the cultures pure and/or at least maintain a pure stock. Both the lactic ensiling culture and the *C. subvermispora* are easy to cultivate. A particular advantage of using microbial pretreatments is that a fraction of the microbial inoculum from the previous batch can be reused to initiate the next round of treatment (back-slopping), similar to classical food fermentations. In conclusion, the presented combination of biopretreatments can be a viable future alternative to high-tech methods, applicable in cases where decreased scale and low plant complexity is more important than relatively long pretreatment time.

## Methods

### Raw material

Wheat straw (*Triticum aestivum* L.), variety hattrick, was harvested and baled in Denmark (N55.67°, E11.92°). This batch of straw was stored in bales indoors at ambient temperature (21 °C) and at constant humidity of ~ 35 % for more than 24 months prior to the experiments (the baled straw was neither chopped further or densified prior to storage). Dry matter content of the stored WS was 90 %.

### Ensiling

Ensiling was carried out on chopped WS (<10 cm) at 35 % weight/weight final TS content, with addition of 6 g xylose per 100 g, in batches of 1.5 kg TS of WS using a vacuum-based plastic bag system (Variovac EK10 vacuum packaging machine, Variovac Nordic A/S, Vejle, Denmark) [9]. For ensiling, each batch of WS was inoculated with commercially available LACTISIL CCM (Chr. Hansen, Hørsholm, Denmark) consisting of freeze dried pure heterofermentative *L. buchneri*. Inoculum size was 8 mg kg^−1^ prepared from a suspension of 0.2 g L^−1^ water and added at 40 mL kg^−1^ WS. The silage incubation time was 4 weeks. Weight loss was measured to calculate TS loss.

### Washing in hot water

Prior to WRF pretreatment a fraction of EWS and a fraction of WS were each washed in 80 °C tap water for 10 min in a biomass to water ratio of 1:15 and then filtered through a 1 mm mesh and dried to ≥20 %TS. Weight loss during the washing was monitored.

### WRF preculture

The fungal strain *Ceriporiopsis subvermispora* CBS 347.63 was obtained from the Fungal Biodiversity Center (CBS) (The Netherlands). The strain was maintained on agar slants at 4 °C. Starting cultures were propagated under aseptic conditions on malt extract agar (MEA) plates (3.36 % w/v, pH 4.7), (Difco™) for 7–10 days at 30 °C.

### WRF pretreatment

50 g TS of WS, EWS, w WS, or w EWS were added to 5 L Erlenmeyer flasks, respectively (the WRF treatments were always done on chopped straw samples [<10 cm]). The moisture content of the biomasses was adjusted in each of the flasks to 20 %TS by Milli-Q water. The flasks were autoclaved at 121 °C for 10 min. The flasks were inoculated by adding 5 plugs of the preculture agar plates (corresponding to 0.5 g inoculated agar) from the newest parts of the colonies. After inoculation, the moisture content was re**-**adjusted in each of the flasks to 20 %TS by Milli-Q water and the flasks were sealed with breathable paper stoppers. The flasks were incubated at 30 °C for 40 days in a controlled humidity chamber at 90 % humidity. The weight-loss was monitored twice a week. Pretreated samples were stored at −20 °C until analysis and further use.

### Total solids (TS) determination

TS were determined by use of the current standard NREL method by Sluiter et al. [38].

### Water extraction

The biomasses (undried) were downsized to <10 mm by use of a Robot Coupe Blixer 3 blender (Robot Coupe, Ridgeland, MS) to ensure homogeneity. Afterwards 0.3–0.4 g TS biomass was extracted in 10 ml MilliQ H_2_O with addition of 10 μl ampicillin (10 mg/ml solution) in order to prevent microbial activity during extraction. The extraction was taking place for 2 h at 25 °C and 150 rpm. Extracts were analysed for mono and oligomeric carbohydrates, acids and phenols. Weight loss after the extraction was determined gravimetrically.

### Analytical method—water-soluble oligomers

Soluble oligomeric carbohydrates in washing waters and water extracts were determined by weak acid hydrolysis. 2.5 ml liquids were mixed with 2.5 ml 4 w/w % H_2_SO_4_ and were autoclaved for 10 min at 121 °C with. Derived sugars were analysed by HPLC as described below.

### Analytical method—sugars and acids

Concentrations of carbohydrates (d-glucose, d-xylose, l-arabinose), organic acids (lactic, formic, acetic, propionic and butyric acids) were quantified by HPLC using a Biorad HPX-87H column (Hercules, CA; USA), RI detector, 63 °C and 4 mM H_2_SO_4_ as eluent, at flow rate of 0.6 ml min^−1^.

### Analytical method—phenols

The total phenols contents were determined spectrophotometrically deploying reaction between phenols and FeCl_3_ as described by Graham [39].

### Determination of structural carbohydrates and lignin

Strong acid hydrolysis was used to measure the carbohydrate and Klason lignin content of the water-extracted biomasses based on the current NREL standard laboratory analytical procedure [38], with the exception that three spiked and three unspiked samples were included in the setup for each of the analysed biomasses (spiked with glucose, xylose and arabinose). Klason lignin was determined from three replicates.

### Enzymatic hydrolysis

The enzymatic convertibility assay based on commercial CellicCTec2^®^ (blend of cellulases) and CellicHTec2^®^ (blend of hemicellulases) (Novozymes A/S, Denmark) was used to determine the efficiency of the pretreatment process. The assay was performed immediately after termination of the pretreatment to take advantage of the enzymes potentially produced during the WRF pretreatment. Enzymatic hydrolysis was performed at 5 % TS content in a total volume of 25 mL using 50 mM citrate buffer (pH 5) and 0.25 mL sodium azide (2 %) at 50 °C shaken at 165 rpm for 72 h. Applied enzyme loadings were 0.11 g enzyme per g TS of CellicCTec2 supplemented with xylanase CellicHTec2 (90:10 ratio-based mass). The enzymatic hydrolysis was performed in triplicates and enzyme blanks were included. Samples were analysed for carbohydrates on HPLC. Enzymatic convertibility was calculated as the converted cellulose divided by the TS content. The measured activity of pure CellicCTec2 and CellicHTec2 were 66 and 29 FPU/ml, respectively (FPU being Filter Paper Units).

### Ethanol fermentation

Fermentations of raw and pretreated biomass were done in 100 mL blue cap flasks containing 5 g TS and 50 ml liquids to a final TS content of 10 % TS. Simultaneous saccharification and fermentation (SSF) with a short pre-liquefaction of 6 h at 50 °C, 165 rpm was applied. As a first step to each flask containing biomass 0.5 mg Tetracycline hydrochloride (Sigma-Aldrich, USA), 475 μL Cellic CTec 2^®^ cellulase complex (Novozymes, Denmark) and 53 μL Cellic Htec 2^®^ endoxylanase (Novozymes, Denmark) and citric acid buffer to a total volume of 46 ml were added. After the liquefaction step, the samples were cooled below 35 °C, and 4 ml citric acid buffer containing 0.1 g TS of *Saccharomyces cerevisiae* yeast (Ethanol Red^®^, Fermentis) was added. The yeast was harvested from an overnight culture (in YPD media) and was washed twice to remove excess growth media. The flasks was flushed with nitrogen, closed with yeast locks and incubated for 144 h at 35 °C, 100 rpm. The fermentations were performed as duplicate for each of the pretreatments (which were done in triplicates), and as triplicates on WS, EWS, w WS, w EWS and a blank containing no biomass.

### BMP determination

For the determination of biogas potentials, triplicate-samples of all biomasses were distributed in 1 l serum flasks (effective volume 1125 ml) in amounts of 1 g volatile solids (VS) per 100 ml active volume. The samples were inoculated with 150 ml of effluent from a lab-scale biogas reactor treating cattle manure and water to a total active volume of 300 ml. For subtraction of biogas produced by the inoculum, flasks containing only inoculum and water were also prepared. The flasks were sealed with rubber septum and metal screw plugs. The samples were incubated at 35 °C for a period of 51 days, hereafter, no more gas production was observed. The CH_4_ production in the flasks was measured by collecting 0.5 ml of headspace gas using a gas tight syringe and analysing the CH_4_ concentration in the sample by gas chromatography (Shimadzu GC-8A, Japan). Measurements were carried out in increasing intervals ranging from 2 days in the beginning to 8 days in the end of the digestion trials. The presented BMP results were based on an average of the last three measuring points, in order to avoid end-point inaccuracy.

### Statistics

The open source software ‘R’ was used for statistical computing. The Analysis of Variance (AOV)-function was used for the one-way ANOVA analyses. Tukey multiple comparisons of means (95 % familywise confidence level) were performed based on the Studentized range statistic and Tukey’s ‘Honest Significant Difference’ method (the TukeyHSD-funcktion in R).

## Additional file

10.1186/s13068-016-0437-x Contents in washing water of wheat straw (w WS) and of ensiled wheat straw (w EWS).

## Abbreviations

WRF: white-rot fungi
WS: wheat straw
E: ensiled
w: washed
F: WRF pretreated
BMP: biomethane potential
TS: total solid
VS: volatile solids
HPLC: high-performance liquid chromatography
GHG: green house gas
SSF: simultaneous saccharification and fermentation
