# Supplementary material for: Combination of ensiling and fungal delignification as effective wheat straw pretreatment
Source: Biotechnol Biofuels. 2016 Jan 22;9:16. doi: 10.1186/s13068-016-0437-x (PMC4728756; doi:10.1186/s13068-016-0437-x)
Supplement: Supplementary file 1 — 10.1186/s13068-016-0437-x Contents in washing water of wheat straw (w WS) and of ensiled wheat straw (w EWS). [file 13068_2016_437_MOESM1_ESM.docx]

Supplementary material

Table S1. Contents in washing water of wheat straw (w WS) and of ensiled wheat straw (w EWS)

| **g/100 g TS** | **Washing water w WS** | **Washing water w EWS** |
| --- | --- | --- |
| **Free Sugars** |  |  |
| Glucose | 0.09 ± 0.01 | 0.00 ± 0.00 |
| Xylose | 0.00 ± 0.00 | 3.11 ± 0.74 |
| Arabinose | 0.00 ± 0.00 | 0.00 ± 0.00 |
| **Oligomers** |  |  |
| Glucan | 0.16 | 0.13 |
| Xylan | 0.34 | 0.00 |
| Arabinan | 0.08 | 0.07 |
| **Acids** |  |  |
| Lactic acid | 0.00 ± 0.00 | 2.25 ± 0.05 |
| Acetic acid | 0.18 ± 0.01 | 1.59 ± 0.02 |
| **Total phenols** | 0.09 ± 0.01 | 0.11 ± 0.01 |
